# Special issue on COVID-19 data integration opportunities and vaccine development strategies

**DOI:** 10.1515/jib-2021-0006

**Published:** 2021-03-22

**Authors:** Jens Allmer

**Affiliations:** Hochschule Ruhr West, Institute for Measurement Engineering and Sensor Technology, Medical Informatics and Bioinformatics, Mülheim an der Ruhr, Germany

Viral infections affect a large part of the human population once or several times each year. Coronaviruses (CoV) are part of the viruses which cause ailments such as the common cold. With SARS-CoV-1, a dangerous variant of CoV caused an epidemic that did not spread worldwide (2002–2004). It has been contained with less than one thousand fatalities (WHO). Another beta coronavirus causing the middle east respiratory syndrome (MERS) broke out about a decade later (2013). While MERS cases are still present in 2021 (most cases reported by Saudi Arabia), the cumulative death toll is below one thousand despite a high case-to-fatality ratio of around 30% [1]. In 2019 SARS-CoV-2 caused a pandemic with abundant worldwide infections and about two million fatalities in early 2021 (http://covid19.who.int). With the SARS-CoV-2 pandemic active for more than one year, vaccines with emergency admittance are being delivered.

**Figure 1:**
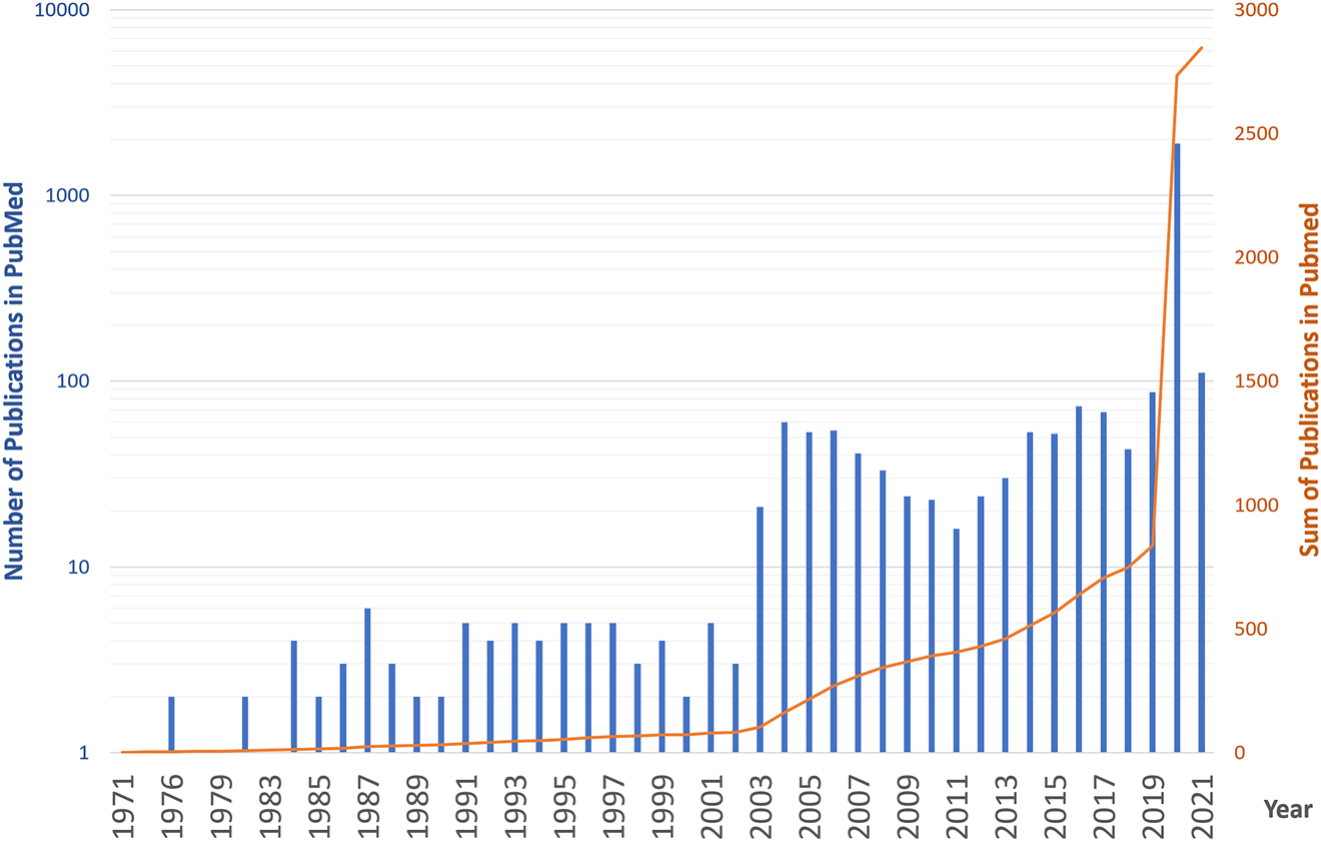
The number of times corona and vaccination occurred in title or abstract in PubMed entries (blue bar graph, logarithmic scaling). The cumulative number of times corona and vaccination occurred in title or abstract in PubMed entries (orange line graph, right *y*-axis).

Interestingly, during 50 years of research on vaccines against coronaviridae, such approaches are only now becoming available (Figure 1). Vaccination of a sufficiently large cohort of individuals to control the pandemic will take long at current vaccination rates. Therefore, it is essential to continue studying SARS-CoV-2 and try additional routes to prevent the virus’s spread or the disease.

Yousef et al. state that testing data is fragmented and not readily available [2]. With a relatively large dataset provided by the Israeli government, they trained a machine-learning algorithm that aided in ranking symptoms, allowing testing prioritization. Demirci and Sacar Demirci show how post-transcriptional gene regulation can be involved in the COVID-19 disease and investigate different miRNAs’ targets and their differential expression [3]. Gültekin and Allmer show how novel information such as RNA binding potential and predicted CoV microRNAs could be incorporated into genome browsers [4]. Such data can help RNA-based drug design. Ahsan et al. tie together many resources with CoV’ information ranging from genomic data to clinical trials [5]. Due to the amount of data generated in the last year, such a resource was desperately needed. OverCOVID will help researchers to find the information they need and may enable integrative studies. Finally, Uttarilli et al. discuss the rapid development of COVID-19 vaccines [6]. Thus, this special issue brings together two applications of COVID-19 data, one visualization of such data, a resource potentially delivering data with integration potential, and a review of vaccine development, which could benefit from the resources mentioned above.
